# MTR3D: identifying regions within protein tertiary structures under purifying selection

**DOI:** 10.1093/nar/gkab428

**Published:** 2021-05-29

**Authors:** Michael Silk, Douglas E V Pires, Carlos H M Rodrigues, Elston N D’Souza, Moshe Olshansky, Natalie Thorne, David B Ascher

**Affiliations:** Computational Biology and Clinical Informatics, Baker Heart and Diabetes Institute, Melbourne, Australia; Structural Biology and Bioinformatics, Department of Biochemistry and Molecular Biology, University of Melbourne, Melbourne, Melbourne, Australia; Systems and Computational Biology, Bio21 Institute, University of Melbourne, Melbourne, Australia; Computational Biology and Clinical Informatics, Baker Heart and Diabetes Institute, Melbourne, Australia; Structural Biology and Bioinformatics, Department of Biochemistry and Molecular Biology, University of Melbourne, Melbourne, Melbourne, Australia; Systems and Computational Biology, Bio21 Institute, University of Melbourne, Melbourne, Australia; School of Computing and Information Systems, University of Melbourne, Melbourne, Australia; Computational Biology and Clinical Informatics, Baker Heart and Diabetes Institute, Melbourne, Australia; Structural Biology and Bioinformatics, Department of Biochemistry and Molecular Biology, University of Melbourne, Melbourne, Melbourne, Australia; Systems and Computational Biology, Bio21 Institute, University of Melbourne, Melbourne, Australia; Computational Biology and Clinical Informatics, Baker Heart and Diabetes Institute, Melbourne, Australia; Structural Biology and Bioinformatics, Department of Biochemistry and Molecular Biology, University of Melbourne, Melbourne, Melbourne, Australia; Systems and Computational Biology, Bio21 Institute, University of Melbourne, Melbourne, Australia; Computational Biology and Clinical Informatics, Baker Heart and Diabetes Institute, Melbourne, Australia; Melbourne Genomics Health Alliance, Melbourne, Australia; Computational Biology and Clinical Informatics, Baker Heart and Diabetes Institute, Melbourne, Australia; Structural Biology and Bioinformatics, Department of Biochemistry and Molecular Biology, University of Melbourne, Melbourne, Melbourne, Australia; Systems and Computational Biology, Bio21 Institute, University of Melbourne, Melbourne, Australia; Department of Biochemistry, University of Cambridge, Cambridge, UK

## Abstract

The identification of disease-causal variants is non-trivial. By mapping population variation from over 448,000 exome and genome sequences to over 81,000 experimental structures and homology models of the human proteome, we have calculated both regional intolerance to missense variation (Missense Tolerance Ratio, MTR), using a sliding window of 21–41 codons, and introduce a new 3D spatial intolerance to missense variation score (3D Missense Tolerance Ratio, MTR3D), using spheres of 5–8 Å. We show that the MTR3D is less biased by regions with limited data and more accurately identifies regions under purifying selection than estimates relying on the sequence alone. Intolerant regions were highly enriched for both ClinVar pathogenic and COSMIC somatic missense variants (Mann–Whitney *U* test *P* < 2.2 × 10^−16^). Further, we combine sequence- and spatial-based scores to generate a consensus score, MTRX, which distinguishes pathogenic from benign variants more accurately than either score separately (AUC = 0.85). The MTR3D server enables easy visualisation of population variation, MTR, MTR3D and MTRX scores across the entire gene and protein structure for >17,000 human genes and >42,000 alternative alternate transcripts, including both Ensembl and RefSeq transcripts. MTR3D is freely available by user-friendly web-interface and API at http://biosig.unimelb.edu.au/mtr3d/.

## Graphical Abstract

**Graphical Abstract:**
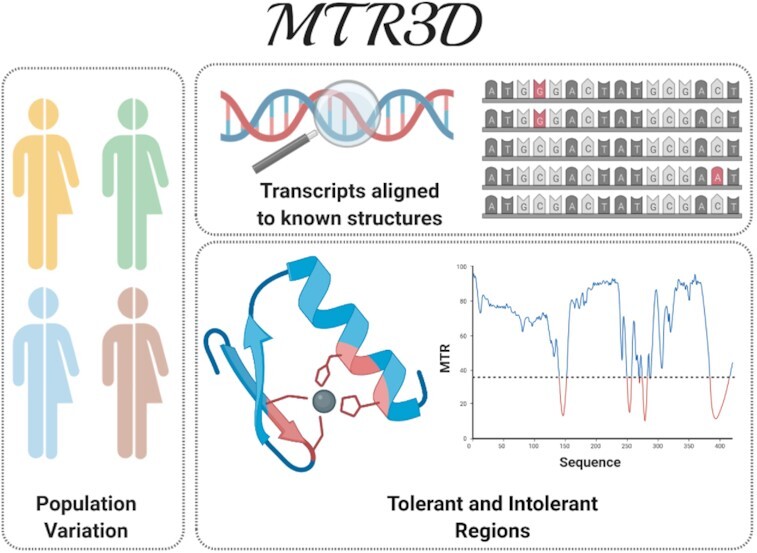
MTR3D.

## INTRODUCTION

Advancements in our ability to distinguish between pathogenic and benign variants using both experimental and computational methods have proven greatly beneficial in our ability to diagnose genetic diseases. *In silico* predictors of deleteriousness have been successfully used to prioritise likely disease-causative variants (1–3), and have proven greatly beneficial in a number of disease cohorts, such as epilepsy, to identify genes enriched for pathogenic variation (4). Despite the accuracy of these methods improving, it remains challenging to identify causative variants due to the diverse effects that a mutation can have on the resulting protein.

Large publicly available datasets of observed variation within the population provide the means to identify regions under purifying selection that are intolerant to genetic change. Several methods have been used to measure this using sequence-based approaches, including RVIS (5), MPC (6) and MTR (7), which have shown that patient-ascertained causative variants are preferentially found within intolerant regions. These provide differing scores depending on whether they are per-gene or regional scores, the sample sizes involved, and the statistical methods used to summarise the degree of depletion. Several tools exist that utilise sequence-based information mapped to protein tertiary structures in order to analyse the impact of mutations (8,9). When examining intolerance scores across a gene's protein tertiary structure, intolerant regions have been observed to cluster within 3D space, but this has not been systematically and comprehensively investigated.

To form a more accurate estimate of missense intolerance, and to systematically investigate genetic intolerance in the tertiary protein space, we introduce the MTR3D, a means of evaluating missense variant deleteriousness based on its spatially measured intolerance. The MTR3D provides both experimental structures from the Protein Data Bank (PDB) and available homology models where a transcript (Ensembl or NCBI RefSeq) could be aligned to a high-quality template.

## MATERIALS AND METHODS

### Data sets

Population variation was combined from gnomAD v2.1.1 (10) (125,748 exomes, 15,708 genomes), gnomAD v3 (76 156 genomes overlapping with gnomAD v2.1.1), the DiscovEHR dataset (11) (50 000 exomes) and UK Biobank (12) (200 000 exomes). Genomic coordinates of DiscovEHR and gnomAD v2.1.1 variants were converted from GRCh37 to GRCh38 reference assembly using LiftOver (13). Variants were then filtered to those single nucleotide variants (SNVs) passing each dataset's quality control filters, annotated using the Variant Effect Predictor (VEP) (Release 101) (14) for positions within Ensembl transcripts and consequence for filtering to synonymous and missense only.

Ensembl transcripts were downloaded from the Ensembl database (v101) (15) using the Bioconductor's biomaRt (16) package. RefSeq transcripts were downloaded from NCBI RefSeq (17) using the biomartr (18) package for NM mRNA transcripts, NP coding sequences and paired with Ensembl transcripts with identical Consensus CDS (CCDS) (19) sequence identifiers. A simulated set of all possible variants was generated by considering every possible single nucleotide substitution (3 variants per nucleotide in the sequence), filtered to missense and synonymous variants, and annotated using VEP to calculate the expected proportion of missense variants.

For validation purposes, ClinVar (20) missense variants were retrieved from the NCBI FTP server and subset based on their labels to pathogenic, likely pathogenic, benign and likely benign variants. The Catalogue of Somatic Mutations in Cancer (COSMIC) v92 (21) variants were downloaded from their website and filtered to confirmed somatic missense variants. The FATHMM SwissProt/TrEMBL disease variants dataset and FATHMM cancer-associated missense variants datasets were also retrieved for additional comparisons (22).

Sequence-based MTR scores can also be viewed in MTR3D, calculated using window sizes of 21, 31 and 41. MTR v1 refers to the MTR scores calculated using gnomAD v1 (23). MTR v2 refers to the current sequence-based MTR scores derived from variation from gnomAD v2.1.1 and v3, UK Biobank and DiscovEHR (7).

### Calculation of the MTR scores across gene transcripts

Missense Tolerance Ratio (MTR) scores were calculated using a sliding window of 21, 31 and 41 codons across each Ensembl and RefSeq transcript by comparing the observed proportion of missense variants to the expected proportion of variants (Equations 1–3).

For a given window $W_i^{H,J}$ and with selected window size $w$, the window is defined as:

(1) $$\begin{eqnarray*}&& {\rm where}\,\, i = {\rm{residue\;position}} \nonumber \\ && H = \max \left( {1,\;i - \frac{{w - 1}}{2}} \right) \nonumber \\ && J = {\rm{min}}\left( {{\rm{transcript\;length}},i + \frac{{w - 1}}{2}} \right) \end{eqnarray*}$$

Within each window, the number of unique missense and synonymous variants are summed at each amino acid position y_i_ for both the observed and expected datasets (Equation 2), and the ratio is taken (Equation 3).

(2) $$\begin{equation*}\begin{array}{@{}l@{}} {y_i} = \mathop \sum \nolimits_{{x_m} \in \left\{ {W_i^{H,J}} \right\}} {x_m}\\ \forall x \in \{ missense\_obs,\;synonymous\_obs,\\ missense\_exp,{\rm{\;}}synonymous\_exp\} \end{array}\end{equation*}$$

(3) $$\begin{equation*}{MTR_i} = \frac{{missense\_ob{s_i}/\left( {missense\_ob{s_i} + synonymous\_ob{s_i}} \right)}}{{missense\_ex{p_i}/\left( {missense\_ex{p_i} + synonymous\_ex{p_i}} \right)}}\end{equation*}$$

### Alignment of transcripts to protein tertiary structures

UniProtKB’s ID mapping table was used to identify pairings between Ensembl and RefSeq transcripts with their corresponding experimental and homology modelled PDB structures and chains (24). Experimentally determined protein structures were downloaded from RCSB Protein Data Bank (25), selecting only the primary biological assembly for each structure. Homology models of Ensembl or RefSeq transcripts were generated using SWISS-MODEL (26) and an in-house homology modelling pipeline using Modeller (27). We considered all potential templates with at least 30% identity for alignments longer than 100 residues and at least 70% identity for alignments shorter than 100 residues. Following minimization in Foldx, the quality of the homology models was assessed using Procheck (28), Molprobity (29) and WHATIF (30). The distribution of QMEAN *Z*-scores for the homology models was examined, revealing that over 76.9% of models have a *Z*-score above –4.0, indicating structural features of the homology models are comparable to what would be expected from high resolution X-ray structures (Supplementary Figure S1).

Ensembl and RefSeq transcripts were aligned to protein tertiary structures in R. Transcripts, metadata and sequences were parsed using data.tables, PDB files were parsed using bio3d (31) and aligned using a ClustalW (32) pairwise alignment via the msa package (33). To take into consideration added and omitted residues (for example unresolved loops) and partial structures (where the experimental structure was generated from a region of the gene, for example a single domain), the alignment was then split where there were gaps of at least three residues. These were then considered separately for congruence of >50% and minimum length of five residues in order to allow unaligned regions to be discarded. 42 312 Ensembl transcripts and 32 845 RefSeq transcripts were aligned to 40 277 unique RCSB PDB structures, 41 861 unique SWISS-MODEL homology models and 43 477 unique homology models generated using Modeller.

### Calculation of the MTR3D scores

Population variation and simulated data of all possible variants, as described above, were mapped to residues within the PDB structure files. At each residue position, in *x*, *y*, *z* coordinates in angströms, missense and synonymous variants were summed based on any residue within a distance of 5, 6 and 8 Å respectively. Proximal residues with at least one atom within each of these spheres were considered (Supplementary Figure S2).

For a given window $W_i^{( {{x_1},{x_2}} ),( {{y_1},{y_2}} ),( {{z_1},{z_2}} )}$ as a sphere of radius $w$, taken from the defined $x,y,z$ coordinates of a residue (Equation 4),

(4) $$\begin{eqnarray*}&& {\rm where}\,\,i = {\rm{residue\;position}} \nonumber \\ && {x_1} = x - w;{x_2} = x + w \nonumber \\ && {y_1} = y - w;{y_2} = y + w \nonumber \\ && {z_1} = z - w;{z_2} = z + w \end{eqnarray*}$$

Observed and expected missense and synonymous variants were summed for each window at each residue *y*._*i*_ (Equation 5).

(5) $$\begin{equation*}\begin{array}{@{}l@{}} {y_i} = \mathop \sum \limits_{{x_m} \in \left\{ {W_i^{\left( {{x_1},{x_2}} \right),\left( {{y_1},{y_2}} \right),\left( {{z_1},{z_2}} \right)}} \right\}} {x_m}\\ \forall x \in \{ missense\_obs,synonymous\_obs,\\ missense\_exp,synonymous\_exp\} \end{array}\end{equation*}$$

(6) $$\begin{equation*}{MTR_i} = \frac{{missense\_ob{s_i}/\left( {missense\_ob{s_i} + synonymous\_ob{s_i}} \right)}}{{missense\_ex{p_i}/\left( {missense\_ex{p_i} + synonymous\_ex{p_i}} \right)}}\end{equation*}$$

The MTR3D was then calculated at each position, considering only positions with at least three observed variants (Equation 6).

MTR3D scores for both ClinVar and COSMIC missense variants were also compared at the different radii of 5, 6 and 8 Å, and separately for experimentally determined and homology modelled structures (Supplementary Figures S3 and S4). This revealed that the 5 Å window size was most informative.

Additionally, both the MTR and MTR3D were calculated for specific populations using a subset of the gnomAD database with sufficient representation of a given population (Admixed American (AMR), Non-Finnish European (NFE) and South Asian (SAS)).

### MTRX consensus score

To assess the extent to which the combination of MTR and MTR3D scores are able to distinguish between pathogenic and non-pathogenic variants, a machine learning model was trained. Uniquely observed missense variants from ClinVar with no conflicting evidence of pathogenicity were assigned the class labels ‘P’, where clinical significance was denoted ‘Pathogenic’ or ‘Likely pathogenic’, or ‘B’ for ‘Benign’ or ‘Likely benign’.

To develop the MTR consensus score, we considered the missense tolerance scores from MTR3D (using a radius of 5 Å), and the previous sequence-based scores from MTR v1 and MTR v2. The performance of each score was considered individually and in combination. In addition, general structural properties including measures of depth, residue solvent accessibility (RSA) and Psi/Phi angles at each residue position, calculated using DSSP 3.0 (34) and Biopython (35), were also considered.

Selecting the most informative features based on predictive performance (Supplementary Table S1), a classifier was generated using Random Forest Classification (n_estimators = 100, max_depth = none, max_features = none, criterion=“gini") with the scikit-learn Python toolkit (36) and evaluated under 10-fold cross-validation, with the best performing model selected based on the area under the ROC curve (AUC) and Matthew's correlation coefficient (MCC). The final classifier MTRX uses MTR3D, MTR v2 21-codon windows, MTR v1 41-codon windows and RSA as evidence to distinguish between variant classes. Only positions with a valid score for these four metrics were given a consensus score.

## WEB-SERVER

We have implemented MTR3D as a user-friendly and freely available web-server application (http://biosig.unimelb.edu.au/mtr3d). The server front end was developed using Materialize framework version 1.0.0, and the back end was built using Python 2.7 via the Flask framework (version 1.0.2). The web-server is hosted on a Linux Server running Apache2.

### Input

MTR3D can be used to either browse a table of the full set of available gene transcripts—PDB structure/chain pairings (Supplementary Figure S5), or to search for a specific gene or transcript directly. Names are not case-sensitive.

**Figure 1.**
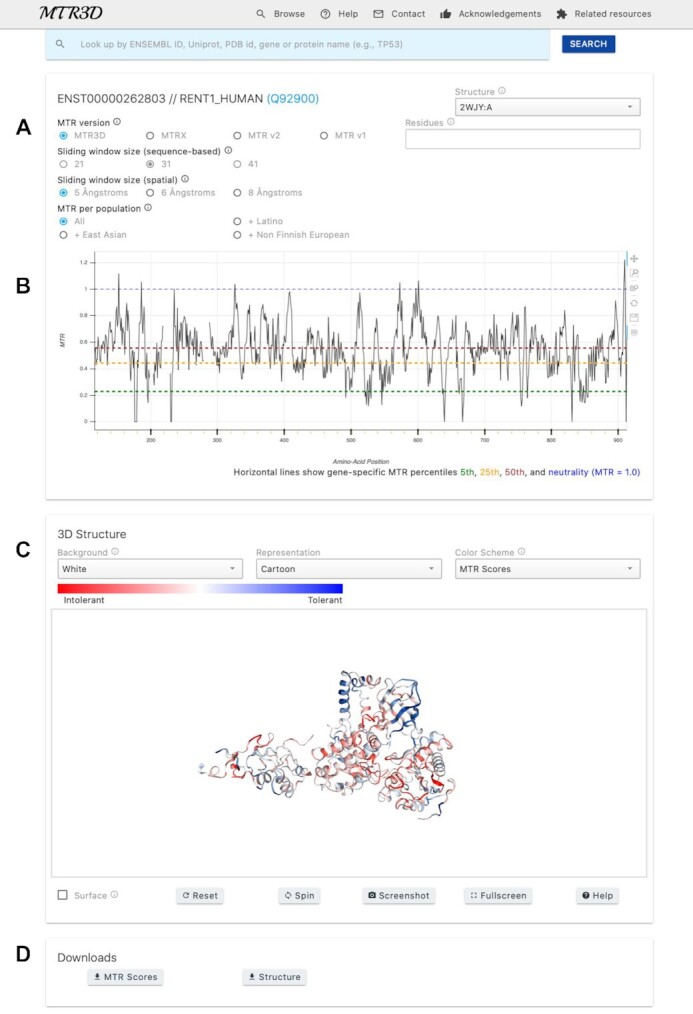
MTR3D viewer page. (**A**) Users may select between different structures and sequence-based, spatial-based and consensus scores for the currently selected transcript. Users may also select between window sizes and population estimates. (**B**) Line graph showing the alignment of scores to the currently selected transcript and structure. Gaps in the plot indicate regions not congruent or not present in the protein tertiary structure. Horizontal lines indicate MTR percentiles for the current transcript at 5th, 25th, 50th and MTR = 1. (**C**) The selected protein structure is displayed and coloured by the currently selected MTR score, where red and blue represent intolerance and tolerance respectively. (**D**) Download links for the MTR scores for the currently selected structure or the currently shown PDB.

On the viewer page (Figure 1) after making a selection, users may select alternate transcripts or alternate structures available for the current transcript or select between different distance calculations from a set of pre-computed options. Sequence-based MTR scores including population-specific MTRs can also be visualised directly on the structure. Users may also submit a protein position or list of protein residues to be highlighted on the structure, based on the transcript's corresponding protein position.

### Output

A line graph using Bokeh is displayed to show the currently selected MTR scores as a 2D representation. This also provides a visualisation of which protein positions of the transcript are present in the currently viewed protein structure. Low scoring MTR3D scores indicate intolerance and purifying selection acting on that region, while high MTR3D scores indicate tolerance and, where MTR3D > 1.0, indicate that variation may be positively selected for in this region.

A viewer to interact with the protein structure is provided, displaying MTR scores mapped onto the structure, where blue coloured regions indicate tolerance and red regions indicate intolerance. The structure can be rotated, zoomed and translated. Individual residue information is displayed when hovering over the structure.

If residues have been selected, a red dot denoting their positions is highlighted on the line graph, and their side chains are displayed in stick format on the structure view.

Both the line graphs and structure representations can be downloaded as image files as currently displayed. A table of MTR scores with alignments between transcript protein positions and structure residue numbers can also be downloaded as a CSV file, as well as the currently displayed PDB structure itself.

## API

An Application Programming Interface (API) implementation is also available for the MTR3D scores for facilitating integration of our method with other systems and applications. Users may query an Ensembl transcript, RefSeq transcript, or HGNC symbol, and may optionally specify a protein position, specific PDB:chain identifier and specific score name. If no specific PDB:chain is supplied, the API will default to the structure with the most coverage for that transcript's alignment to the structure. If no protein position is supplied, the API will return all scores across the currently selected structure. If a specific score is selected, the API will only return values for that score. Results are returned as a JSON object.

### Datasets

A bulk download is available via the web-server to download the full set of scores for Ensembl and RefSeq transcripts mapped to the experimental and homology structures. ClinVar disease variants, COSMIC somatic variants and DiscovEHR population control variants used for validation are also available for download via the web-server.

## VALIDATION

### Performance on disease-ascertained variants

MTR3D was assessed for its ability to differentiate pathogenic from non-pathogenic variants by comparing MTR3D scores across the ClinVar dataset. For each ClinVar gene transcript, a single protein structure with the greatest number of matching residues was selected, then ClinVar variants were filtered to uniquely observed variants by removing duplicate observations in order to prevent bias towards gene symbols with many transcripts or overrepresented variants. Note that validation could only be performed on ClinVar genes with a valid structure (2752 experimental structures, 6333 homology modelled structures). Performance of experimentally determined protein structures was assessed separately to the homology modelled structures to assess whether both show similar enrichment of pathogenic variants within intolerant regions (Supplementary Figure S3).

**Figure 2.**
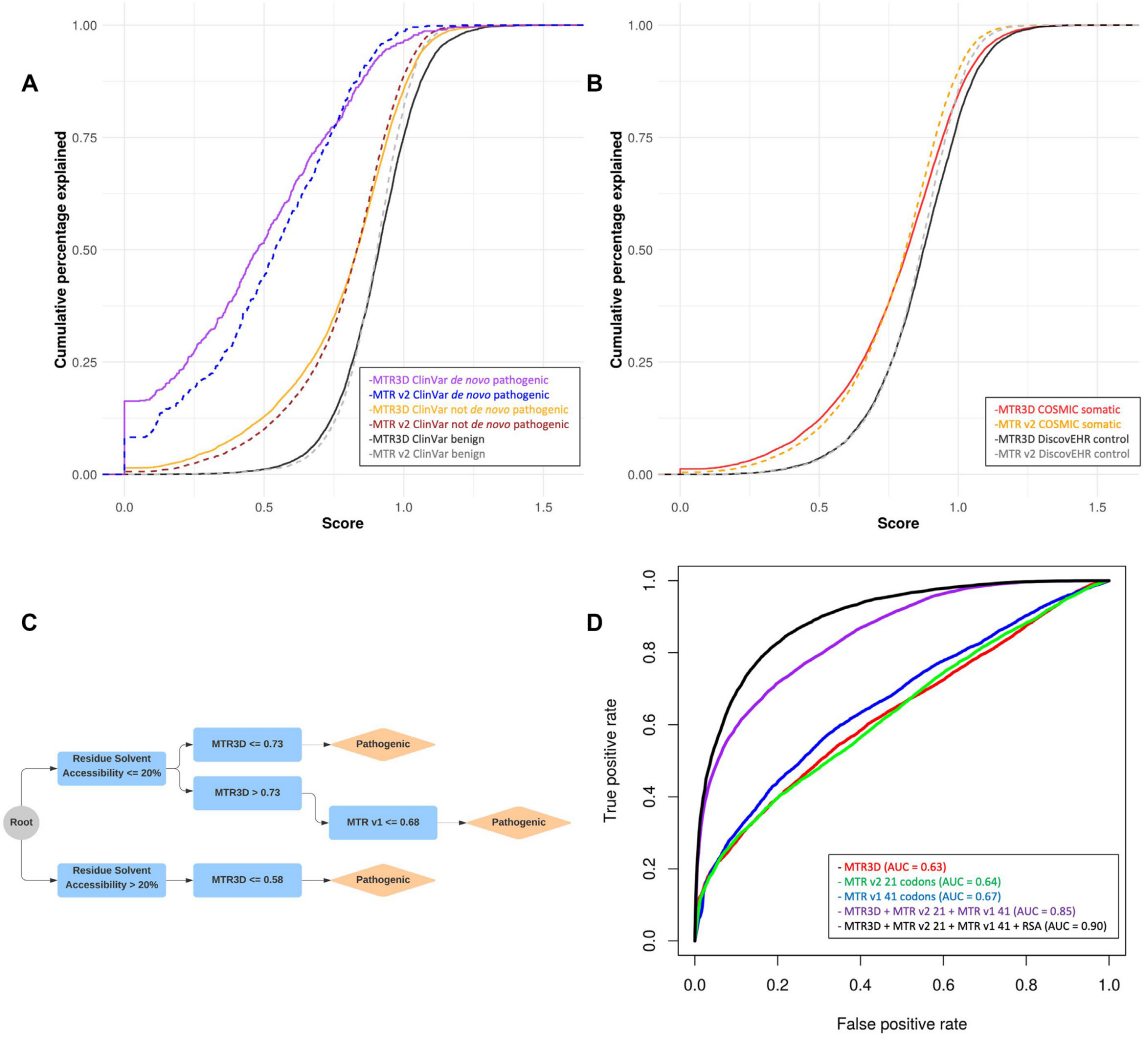
Performance of MTR3D and consensus score on identification of disease and cancer-ascertained variants. Comparison of the spatial- and sequence- based MTR scores using disease-associated variants. (**A**) Cumulative distribution graph comparing MTR3D (5 Å) and MTR v2 (31 codons) in ClinVar *de novo* pathogenic missense variants (purple, blue respectively), ClinVar not *de novo* pathogenic missense variants (orange, brown respectively) and ClinVar benign missense variants (black, grey respectively). (**B**) Cumulative distribution graph comparing COSMIC somatic missense variants MTR3D (5 Å) scores (red), MTR v2 (31 codons) scores (orange), with DiscovEHR population missense variants observed within the same genes (black, grey respectively). (**C**) Decision tree representation of the most informative scores used in the generation of the consensus metric calculated using a Random Forest model. Cut-offs were determined based on 10-fold cross-validation. (**D**) Area under the Curve (AUC) plot showing classification specificity/sensitivity for MTR3D (5 Å) (red), MTR v2 21 codons (green), MTR v1 41 codons (blue), MTR consensus using MTR3D (5 Å) + MTR v2 21 + MTR v1 41 (purple) and with RSA included (black).

Intolerant regions were found to be significantly enriched for ClinVar non *de novo* pathogenic variants (*n* = 14 547) and *de novo* pathogenic variants (n = 725) than benign variants (n = 7,086) for both experimentally determined and homology modelled structures (Figure 2A; Mann–Whitney *U* test *P* < 2.2 × 10^−16^ and *P* < 2.2 × 10^−16^, respectively). At a MTR3D (5 Å) <0.5, which we consider to be intolerant, 537 of 725 ClinVar *de novo* pathogenic and 5030 of 14 547 ClinVar non *de novo* pathogenic variants were observed, while only 856 of 7086 benign variants were found here. The MTR3D scores was further assessed using the FATHMM SwissProt/TrEMBL training dataset and found to perform similarly (Mann–Whitney *U* test *P* < 2.2 × 10^−16^).

### Performance on cancer-ascertained variants

COSMIC unique somatic missense variants from cancer samples were compared with DiscovEHR population variants to determine whether there is significant enrichment of COSMIC variants within intolerant regions compared with standing variation within the population (Figure 2B). We defined a proposed cutoff of 0.75 to denote intolerance, however the ideal cutoff will vary depending on the gene under investigation. Over two thirds of COSMIC variants (18 981/27 520) were found to have a MTR3D <0.75. A significant enrichment was found in both experimentally determined and homology models for COSMIC variants (Supplementary Figure S4; Mann–Whitney *U* test *P* < 2.2 × 10^−16^ and *P* < 2.2 × 10^−16^, respectively). Using the FATHMM cancer-associated training dataset, we find similar enrichment for cancer-associated variants within intolerant regions (Mann–Whitney *U* test *P* < 2.2 × 10^−16^).

Interestingly, when we compared the intolerance scores of variants in tumour suppressor (*n* = 116 genes) and oncogenes (*n* = 91 genes) separately, while background control variation did not reveal any significant difference, cancer-ascertained variants in oncogenes were associated with significantly lower MTR3D scores than those in tumour suppressors (Supplementary Figure S6). This is likely due to the larger effect of purifying selection of dominant variants.

### Performance of the MTRX consensus score

A consensus score, MTRX, was built using the MTR3D scores, together with sequence-based MTR scores and general structural properties, using the ClinVar database (*n* = 23 406 variants). The MTRX represents a likelihood of a variant being pathogenic [0–1]. The distribution of MTRv1, MTRv2, MTR3D and RSA across the dataset shows clear differences between benign and pathogenic variants (*P*-value < 0.0001, Supplementary Figure S7), and interestingly there is not a strong correlation between the spatial and sequence based scores (Supplementary Figure S8). The overlap in intolerant regions between the spatial and sequence based scores, indicated that while there was significant agreement, over 18% of the intolerant regions under selective pressure were identified by only the spatial based scores, in particular in sequence based windows with limited data (Supplementary Figure S9).

Table 1 shows the predictive performance of individual scores and their combination under 10-fold cross validation. Individually, MTR scores achieved AUCs of 0.63 (MTR3D; 5 Å), 0.64 (MTR v2; 21 codons) and 0.67 (MTR v1; 41 codons), respectively (Figure 2D). While individual features only presented a modest ability of distinguishing between pathogenic and benign variants, a significant improvement in performance (*P*-value < 0.001) is observed when scores are combined in a consensus, achieving an AUC of 0.85 and MCC of 0.49, demonstrating the complementary nature of the scores. Performance is further improved when structural properties (residue relative solvent accessibility) is also considered (Figure 2D; AUC of 0.90 and MCC of 0.62). An analysis of feature importance also showed a high level of complementarity between MTR scores and the selected structural property (Supplementary Figure S10).

**Table 1.** Predictive performance of MTRX consensus scores on ClinVar variants

Score	TP rate	FP rate	Precision	Recall	AUC	MCC
**MTR3D 5 Å**	0.64	0.57	0.60	0.64	0.63	0.10
**MTRv2 (21 codons)**	0.64	0.55	0.60	0.64	0.64	0.12
**MTRv1 (41 codons)**	0.65	0.49	0.63	0.65	0.67	0.17
**MTR3D + MTRv2 + MTRv1**	0.77	0.30	0.77	0.77	0.85	0.49
**MTRX**	0.83	0.22	0.83	0.83	0.90	0.61

Exploring the learned trees further, we observe that the top of the majority of the decision trees uses as first branching point an RSA of 20.7% (Figure 2C). Interestingly, this is consistent with general thresholds for considering residues as either buried (RSA < 20%) or exposed (RSA > 20%) (37,38). For buried residues, MTRX considered a variant pathogenic if the MTR3D score was below 0.73 or the MTRv1 score <0.68 (Figure 1A). For exposed residues, variants were considered pathogenic if their MTR3D score was below 0.58, indicating the need for stronger evidence of intolerance to label exposed residues as pathogenic than buried residues. These two simple rules covered over a quarter of the data.

## CONCLUSION

The MTR3D application provides an intuitive and programmable interface to explore intolerance and purifying selection within protein tertiary structures and across the flat sequence. The MTR3D and MTR consensus scores are versatile metrics to assess the likelihood of pathogenicity in patient-ascertained variants, as well as measures to identify novel important regions within protein structures that may be overlooked by traditional metrics.

## DATA AVAILABILITY

MTR3D scores and data are freely available either via the user-friendly web interface, as a bulk download or through an API for programmatic access at http://biosig.unimelb.edu.au/mtr3d No login or license is required.

## Supplementary Material

gkab428_Supplemental_File
