# Supplementary material for: MTR3D: identifying regions within protein tertiary structures under purifying selection
Source: Nucleic Acids Res. 2021 May 29;49(W1):W438–45. doi: 10.1093/nar/gkab428 (PMC8265191; doi:10.1093/nar/gkab428)
Supplement: gkab428_Supplemental_File [file gkab428_supplemental_file.docx]

**SUPPLEMENTARY DATA**

**MTR3D - Identifying regions within protein tertiary structures under purifying selection**

Michael Silk^1,2,3^, Douglas Pires^1,2,3,4^, Carlos M. Rodrigues^1,2,3^, Elston N. D’Souza^1,2,3^, Moshe Olshansky^1^, Natalie Thorne^5^, David B. Ascher^1,2,3,6,*^

^1^ Computational Biology and Clinical Informatics, Baker Heart and Diabetes Institute, Melbourne, Australia

^2^ Structural Biology and Bioinformatics, Department of Biochemistry and Molecular Biology, University of Melbourne, Melbourne, Melbourne, Australia

^3^ Systems and Computational Biology, Bio21 Institute, University of Melbourne, Melbourne, Australia

^4^ School of Computing and Information Systems, University of Melbourne, Melbourne, Australia

^5^ Melbourne Genomics Health Alliance, Melbourne, Australia

^6^ Department of Biochemistry, University of Cambridge, Cambridge, UK

^*^ To whom correspondence should be addressed D. B. A. Tel: +61 90354794; Email: [david.ascher@unimelb.edu.au](mailto:david.ascher@unimelb.edu.au).

**
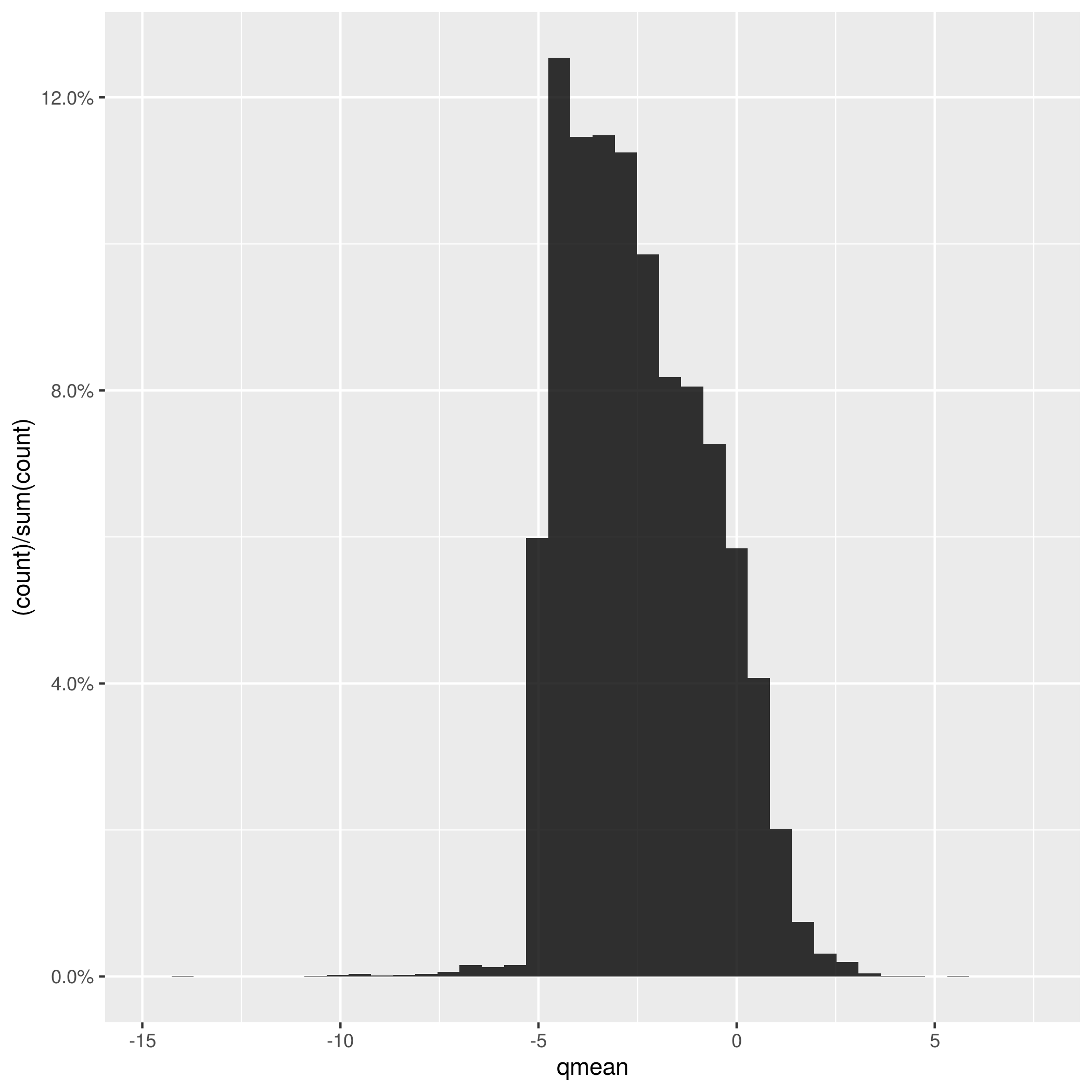
**

**Figure S1: Homology modelled QMEAN Z-scores.** Histogram of Z-scores for included homology models. Scores below -4 are considered lower quality.

**
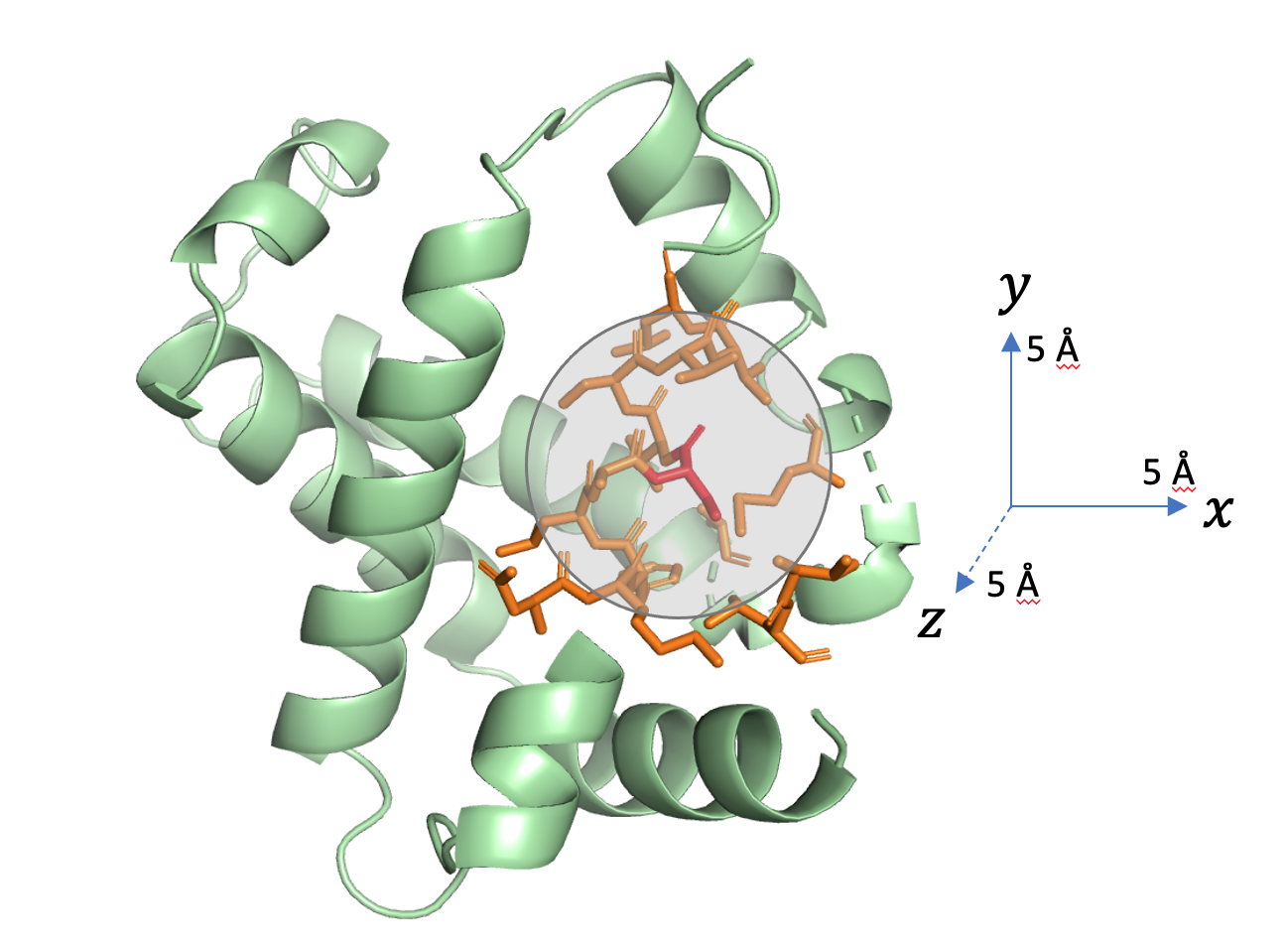
**

**Figure S2: Schematic of spatial windows.** Example window calculation using window size 5Å for residue 131 (red) in the experimental structure 1a01.pdb, chain A. Residues with at least one atom within the defined window are selected (orange).


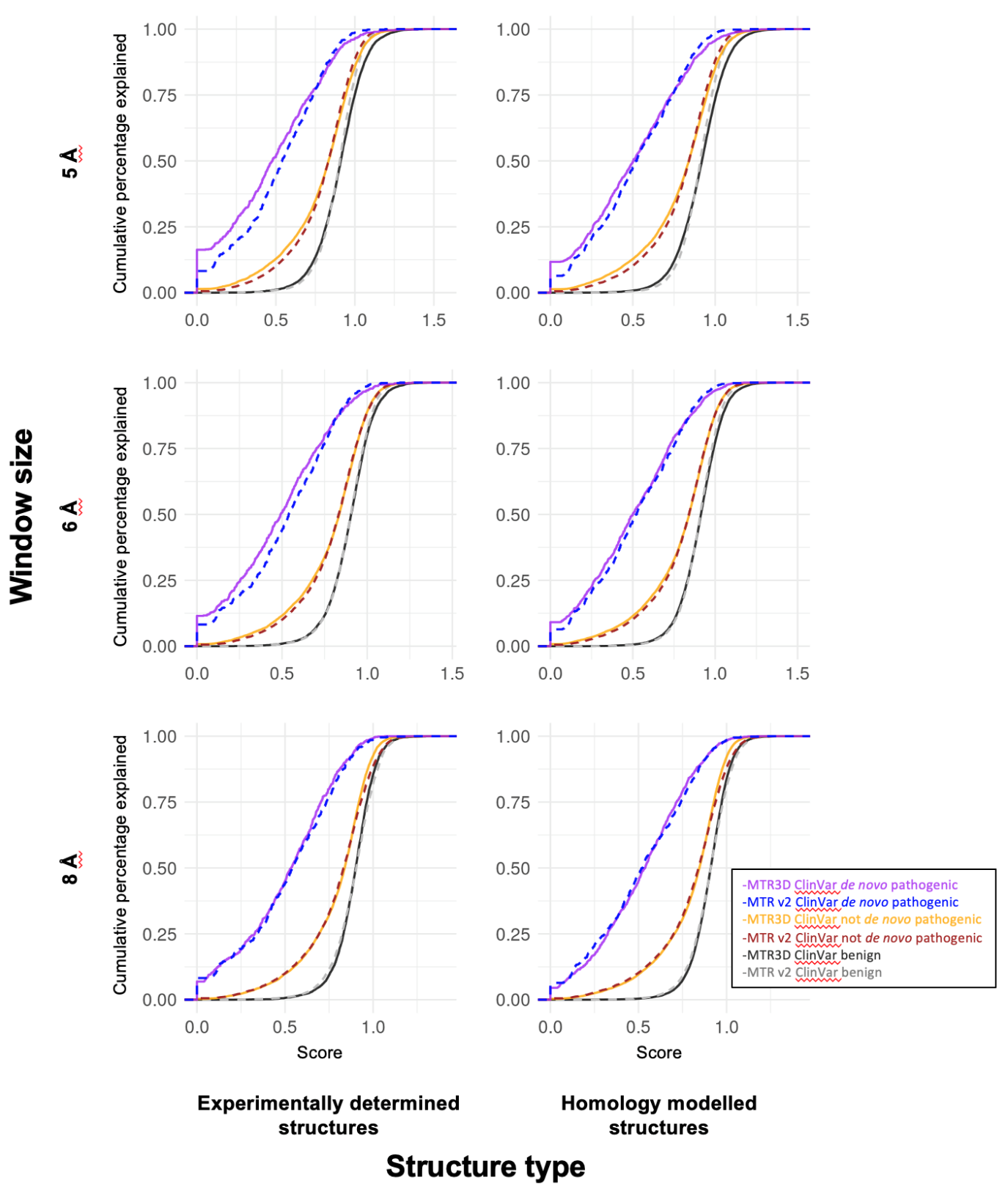


**Figure S3: Performance of MTR3D at different window sizes on the identification of disease-associated missense variants.** MTR3D scores calculated at different spatial window sizes of 5 Å, 6 Å and 8 Å, and separately compared between the experimentally determined and homology modelled structures, for ClinVar *de novo* pathogenic variants (purple), ClinVar not *de novo* pathogenic variants (orange) and ClinVar benign variants (black). These are also compared with MTR v2 (31 codons) scores (blue, brown, grey respectively).


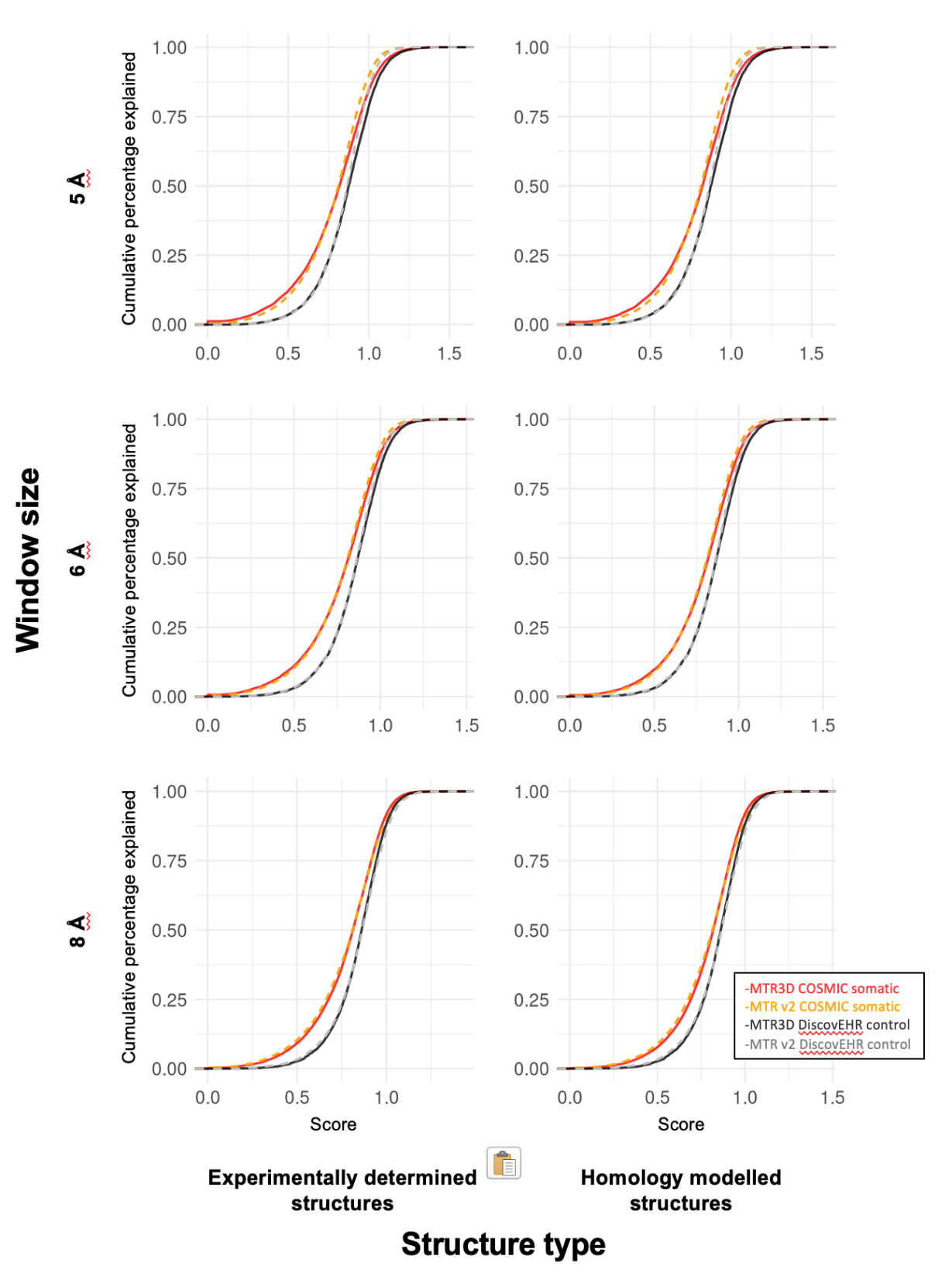


**Figure S4: Performance of MTR3D at different window sizes on the identification of cancer-ascertained missense variants.** MTR3D scores calculated at different spatial windows of 5 Å, 6 Å and 8 Å, and separately compared between experimentally determined and homology modelled structures, for COSMIC missense variants (red) and DiscovEHR population missense variants (black). MTR v2 (31 codons) scores are also compared (orange and grey respectively).


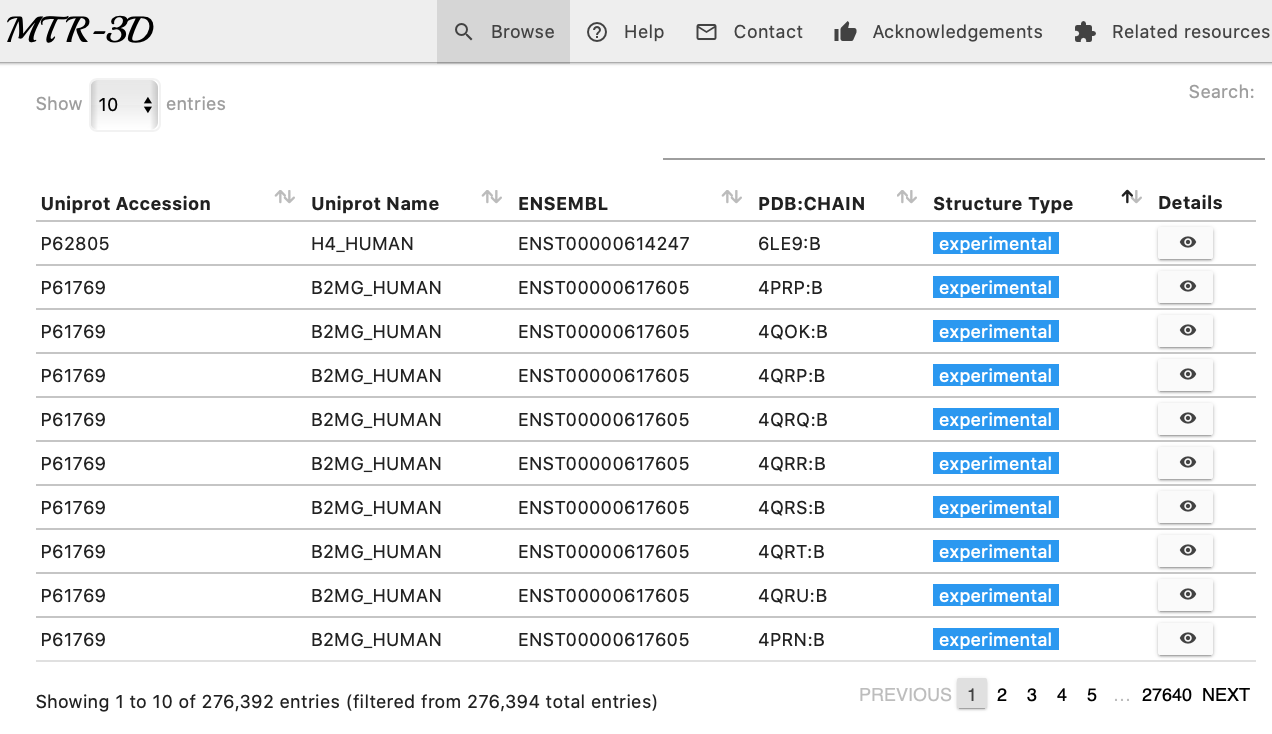


**Figure S5: MTR3D browser page.** Browser page for querying HGNC gene symbols, UniProt accessions, Ensembl and RefSeq transcripts. Results can be sorted by clicking on a column name. Clicking on the eye icon will redirect to the Viewer page.


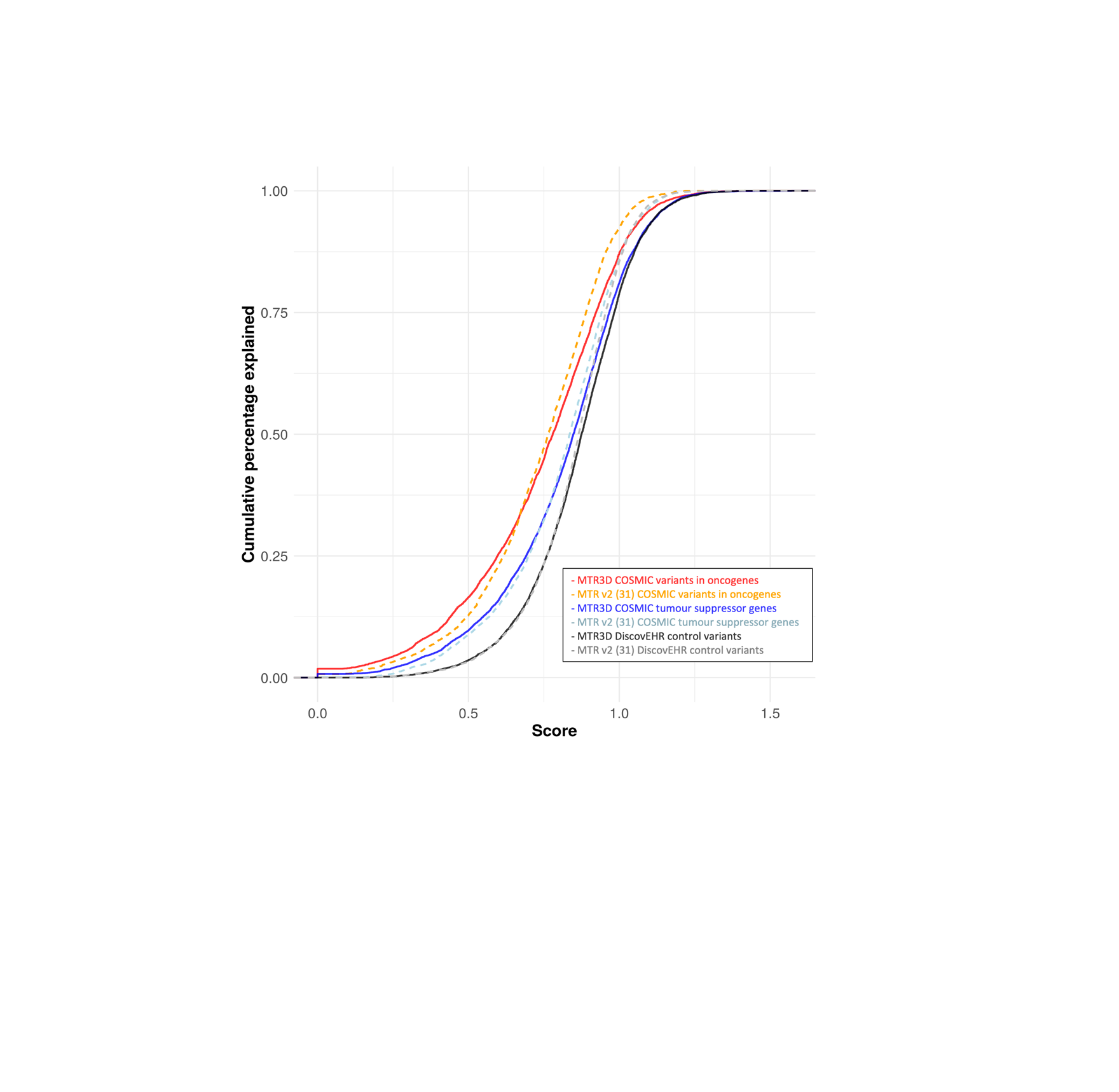


**Figure S6: MTR3D and MTR scores in COSMIC oncogenes and tumour-suppressor genes.** Cumulative distribution graph showing MTR3D 5 A and MTR v2 31 codons scores in COSMIC oncogenes (red and orange respectively) and in COSMIC tumour-suppressor genes (blue and light-blue respectively), and compared with DiscovEHR population variants (black and grey respectively).


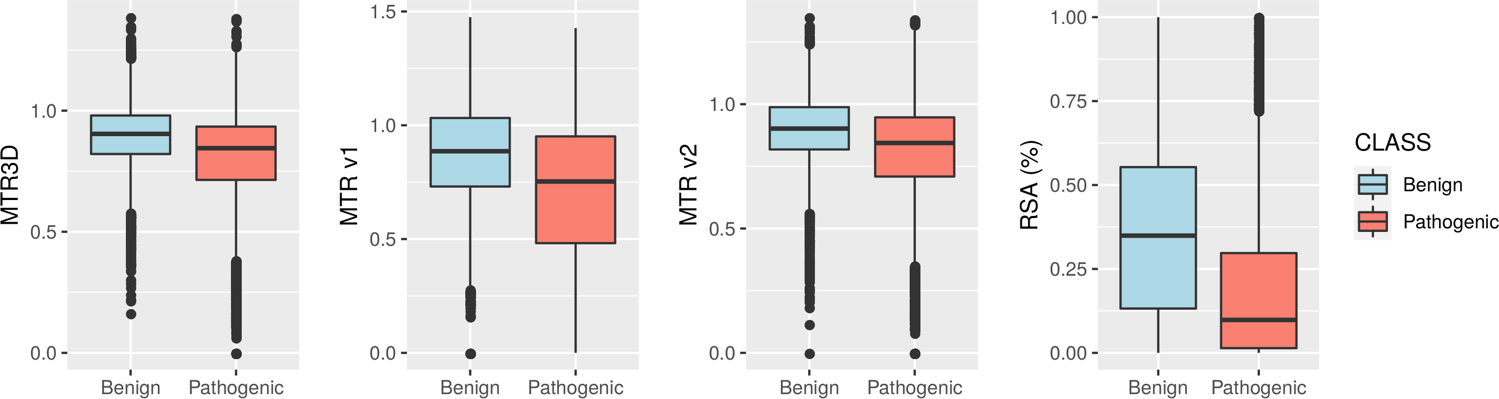


**Figure S7: Distributions of MTR scores in disease-associated genes.** MTR3D and sequence-based MTR scores are compared for ClinVar pathogenic missense variants (red) and ClinVar benign missense variants (blue).


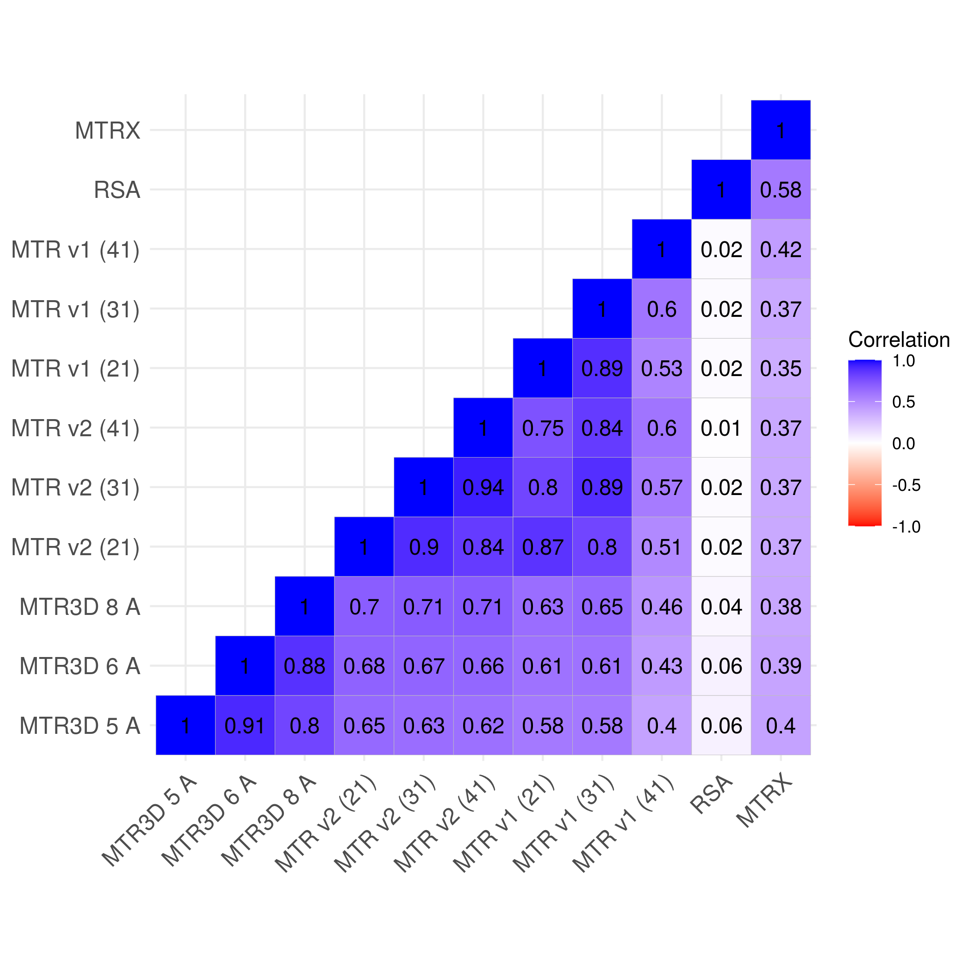


**Figure S8: Correlation between scores**. Pearson’s Correlation between MTR scores, Residue Solvent Accessibility (RSA) and MTRX consensus score for ClinVar variants. MTRX scores (higher scores indicate likely pathogenic) were inverted for direct comparison with MTR scores (lower scores indicate intolerance).


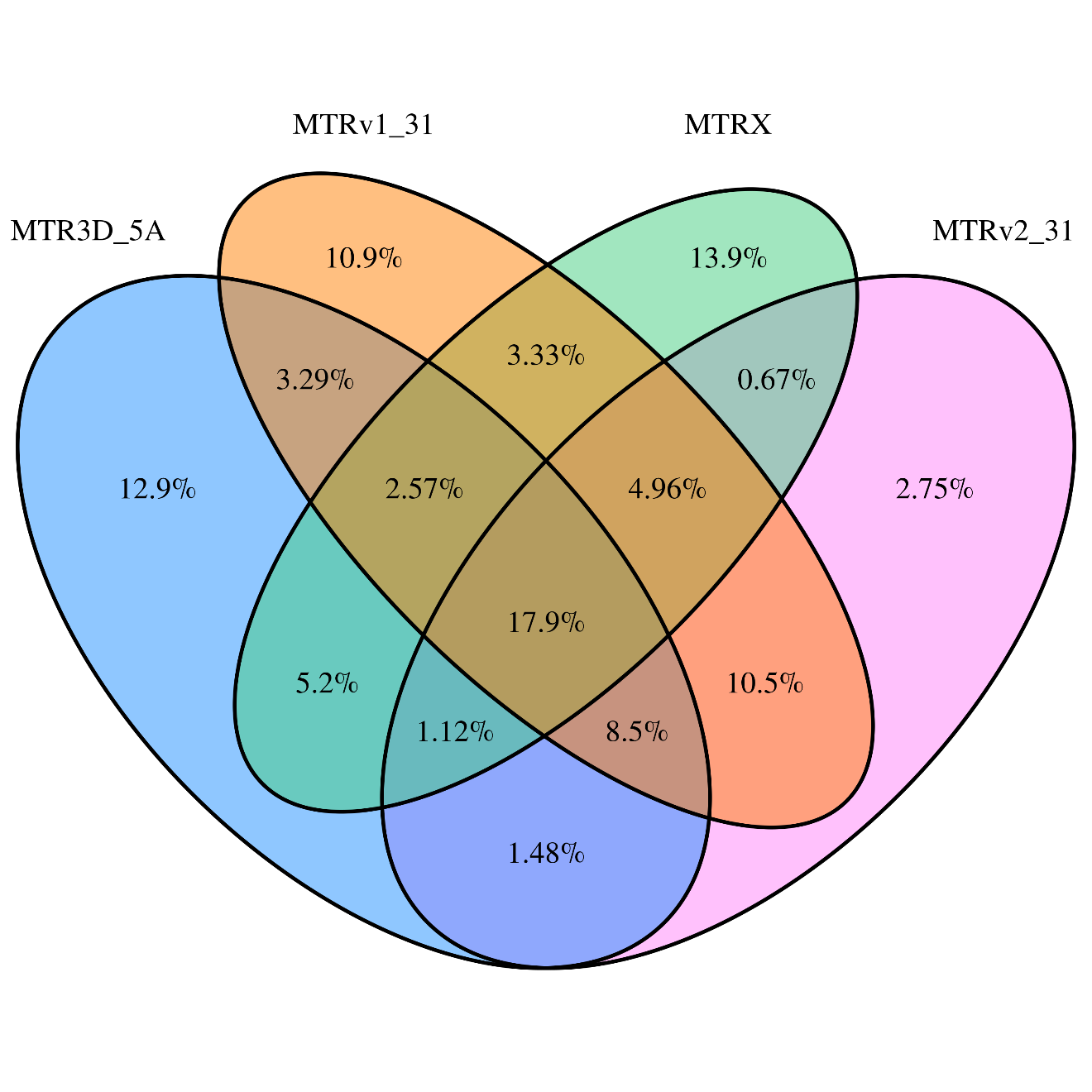


**Figure S9: Agreement between scores across the full dataset.** All positions mapped to all structures for Ensembl transcripts with no missing values (n = 50,770,176) were queried for whether they were scored as intolerant by MTR3D (5 Å), MTR v1 (31 codons), MTR v2 (31 codons) using a cutoff of 0.75, corresponding with the 25^th^ percentile of scores. For comparison, MTRX was considered intolerant using a cutoff of 0.82 corresponding with its 25^th^ percentile.


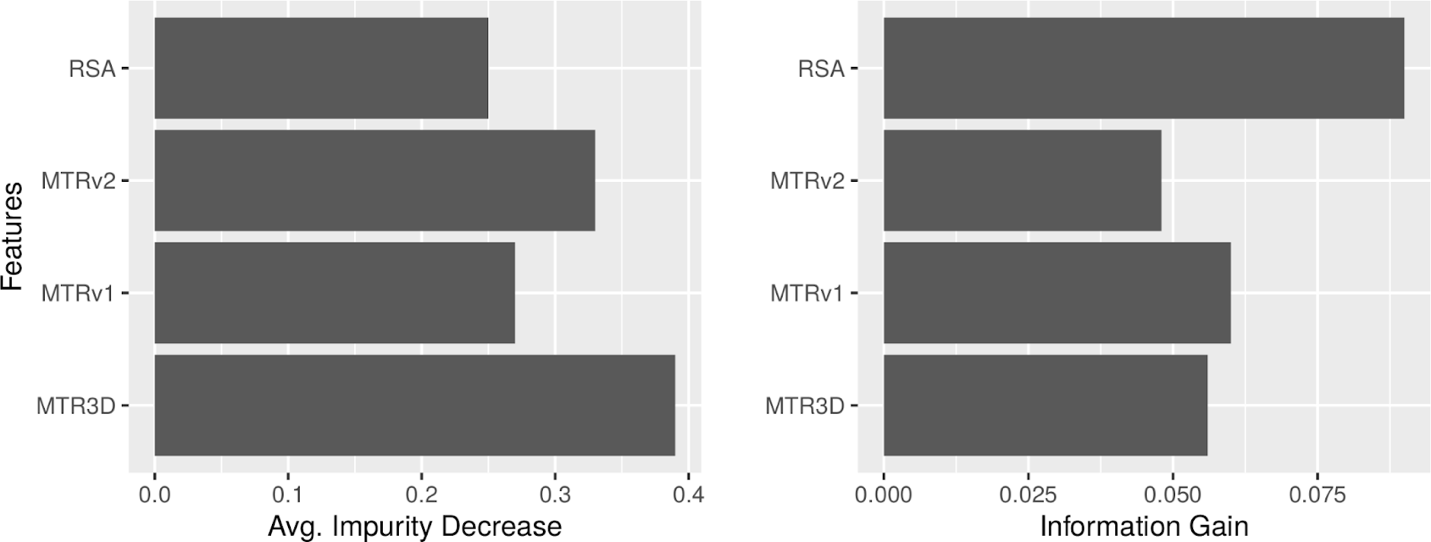


**Figure S10.** Analysis of feature importance for the MTRX consensus score. The plots show importance based on average impurity decrease (obtained using Random Forest Classifier) and Information Gain (right), highlighting a relatively similar contribution from all features employed.

**Table S1: Predictive performance of different consensus scores**

| **Predictive model** | **Accuracy** | **AUC** |
| --- | --- | --- |
| MTRX with RSA | 0.83 | 0.90 |
| MTRX with Residue Depth | 0.81 | 0.89 |
| MTRX with Dihedral angles | 0.79 | 0.87 |
